# Understanding how, for whom and under what circumstances telecare can support independence in community-dwelling older adults: a realist review

**DOI:** 10.1186/s12877-024-05650-6

**Published:** 2025-01-27

**Authors:** Lauren Fothergill, Niall Hayes, Yvonne Latham, Jenny Hamilton, Saiqa Ahmed, Carol Holland

**Affiliations:** 1 Lancaster University, Lancaster, UK; 2 University of Leeds, Leeds, UK; 3 National Institute for Health and Care Research, London, UK

**Keywords:** Telecare, Older adults, Independent living, Health and well-being

## Abstract

**Background:**

There is substantial interest among policy makers in using telecare to support independence in older adults. However, research on how telecare can be most beneficial in promoting independence is limited. This realist review aimed to understand the contexts in which telecare can support independence and for whom, to aid older people in remaining at home.

**Methods:**

This realist review is consistent with the RAMESES quality and reporting standards. We followed a five-step process to conduct the review: (1) locating existing theories and concepts, (2) searching for evidence (3), selecting data, (4) extracting data, and (5) synthesising data. We analysed 32 studies published between 2004 and 2023 to identify core mechanisms of how telecare may lead to positive or negative impacts in the form of context-mechanism-outcome (CMO) configurations. CMOs were grouped into overall domains and contributed to an overall programme theory of how telecare works.

**Results:**

Four key domains across 12 CMO configurations were identified, which suggest how telecare can support older adults in living independently (1). Telecare services should support older adults’ goal of staying at home by providing reassurance of help in an emergency and aid in detecting age-related deterioration (2). Telecare that supports autonomy by enabling choice over technological resources may support self-reliance and control over one’s life, including choosing the level of monitoring, freedom to call for help if needed, and the ability to customise technology to suit needs (3). Telecare that enables connections to existing or new social networks may reduce loneliness and social isolation for those who lack social resources. Finally (4), telecare must integrate into everyday life by fitting people’s existing context, skills, resources, and identity. To improve telecare implementation, consideration must be given to these mechanisms; otherwise, interventions risk being abandoned or underutilised and, as a result, may not adequately support older adults to remain living at home safely, creating a false sense of security.

**Conclusions:**

Assessments of an individual’s needs and preferences should be carried out to ensure that telecare enables autonomy, supports the goal of remaining at home, facilitates connections to social support, and promotes integration into everyday life.

**Study registration:**

PROSPERO CRD42021292384.

**Supplementary Information:**

The online version contains supplementary material available at 10.1186/s12877-024-05650-6.

## Introduction

Globally, populations are ageing, with data predicting that by 2050, one in six people will be aged older than 65 in the world [1]. An increased prevalence of chronic illnesses in older populations can result in an increased need for social care support [2]. For example, in the United Kingdom (UK), an estimated 2.2 million people over 65 years of age require support with at least one activity of daily living (ADL) [3]. To reduce the pressure on health and social care [4], the home environment is becoming an increasingly important setting for the delivery of social care interventions. Most older adults wish to preserve their independence and to be able to remain living in one’s own home [5]. Maintaining independence can refer to being self-reliant, maintaining a sense of control, having social relationships and obtaining help if needed [5, 6]. Telecare use is promoted to support people in living independently [7, 8]. It is characterised by various forms of monitoring technologies that manage risks associated with independent living; examples include pendant alarms, fall detector sensors [9], and other behavioural and environmental sensors [2]. Telecare differs from telehealth in that telehealth involves remote medical monitoring of individuals at home by healthcare professionals or clinicians. Telehealth technologies track vital signs, such as blood pressure after a stroke, to promote self-care while allowing healthcare providers to monitor patients from a distance [10]. Telecare has the potential to support older adults in living at home by detecting potential accidents, injuries, and poor health, thus facilitating a safe environment and protecting individuals from avoidable harm [11].

An estimated 1.7 million people currently use telecare in the UK, mostly older adults [12]. Telecare is purported to support independent living by reducing hospital admissions, delaying institutionalisation, and supporting well-being and quality of life [13, 14]. However, the evidence supporting these claims is mixed. While some studies have shown that older adults view telecare as beneficial for enhancing health and well-being by providing a sense of security, reducing fear of falls and increasing confidence [15, 16], other studies raise concerns among older adults regarding privacy issues associated with telecare [17, 18]. This potential lack of privacy could reduce autonomy and overall well-being [19]. Despite these findings, telecare continues to be offered by local authorities across England as a way of preventing or delaying the need for care [20].

Systematic reviews have shown evidence of the efficacy of telecare and monitoring technologies [21, 22]; however, these reviews also highlight that there is no *‘one size fits all’*, and future research should focus on *how* to adapt technology to the individual needs and resources of older adults. However, recent reviews have not attempted to unpack the underpinning generative forces required for telecare to be effective in supporting health and well-being for different individuals. Woolham et al. (2019) suggest that the locus of the problem is not the technology itself but how it is implemented and utilised [23]. Despite the varying contexts in which telecare is used, the technology is often deployed in a ‘plug and play’ manner, which refers to the idea that telecare can be easily installed and used without needing adaptation to the specific needs of the user, which may not be suitable for some individuals [10, 24, 25]. Although research to date has offered insights into the potential efficacy of telecare devices, there has been little research into developing and refining theories on how telecare can support independence, health, and well-being and under what conditions, which would allow for better targeting of telecare to contexts where it is likely to be effective in promoting health. Given that older adults are not homogenous, telecare may support people in different ways or may not be able to meet everyone’s needs.

A realist approach enables the exploration of what works for whom and how. This methodology is well suited for addressing the recognised need for further research on matching telecare devices to individual needs and resources. Therefore, the aim of this realist review is to develop and refine theory about how telecare can support independent living in older adults, for whom and under what conditions.

## Methods

### Study design

A realist review was conducted that explored how telecare might support older adults who wish to use technology to support their independence. A realist review is a theory driven approach, which aims to identify the underlying mechanisms that function within specific contexts to produce particular outcomes, helping to understand how an intervention works [26]. The RAMESES quality and reporting standards for reporting realist reviews were followed [27]. Programme theories are formed during a realist review and are typically presented as evidence-based context-mechanism-outcome (CMO) configurations. These describe the contexts in which certain mechanisms, often hidden, elicit specific outcomes from using telecare. A glossary of terms is provided in Table 1.

**Table 1:** Glossary of terms

Context (C)	Context refers to the conditions in which the intervention operates. Context can refer to the individual taking part in the programme, or wider cultural, economic, and societal settings for programmes [28].
Mechanism (M)	Explains how an intervention works through defining underlying processes, which operate in contexts to produce certain outcomes. A mechanism includes the *resources* offered through an intervention (for example, help in an emergency) but also the individual’s reaction and *response* to these resources (for example, engagement and motivations from the individual) [29].
Outcome (O)	Outcome refers to the observed products following engagement with an intervention (intended or unintended) [26].
CMO configuration	A context-mechanism-outcome configuration (CMO) is a heuristic used to theorise how an intervention works, for whom and in what circumstances. CMOs may focus on a particular aspect of an intervention, or the intervention generally [30].
Initial programme theory (IPT)	IPT refers to potential ideas to how and why an intervention may work. IPTs will include potential contexts, mechanisms, and outcomes of interest to test in further empirical research.
Middle range theory (MRT)	MRT is a developed theory that can be used to explain the cause of outcomes for interventions. ‘Middle range’ means that the theory can be tested with observable data and is not theorising an abstract social force [26].
Programme theory	This explains how the intervention may work. In this review, individual CMO configurations represent individual programme theories, explaining specific components of the intervention. We also present an overarching programme theory, which summarises how telecare may work to support independent living in older adults.

This review followed Pawson’s five stages for conducting realist reviews: 1) locating existing theories and concepts, 2) searching for evidence, 3) selecting data, 4) extracting data and 5) synthesising data [31]. The research team and two National Institute for Health and Social Care Research (NIHR) public advisors with experience caring for older adults were involved in the process.

#### Step 1: locate existing theories and concepts

This research began with an exploratory search to explore initial programme theories (IPTs) about how telecare might work by identifying models or theories associated with supporting the health and well-being of older adults. We initially drew upon Baltes & Baltes (1990) well-established model of selective optimisation with compensation (SOC) [32], which involves everyday adaptations that older adults engage in to maximise gains and minimise losses in response to age-related challenges [33]. When older adults are faced with age-related challenges, individuals select a goal to focus on, optimise their resources (by acquiring and refining resources), and compensate existing resources for alternative ones to pursue their goal [34]. The SOC model has been used across the literature to understand how older adults use various resources to maintain health and well-being. This was considered a well-suited theoretical starting point, given that telecare uptake is often utilised to minimise functional losses (through falls, accidents, and other health-related risks) by ensuring safety at home [35]. However, the SOC model was not specific to the programme architecture of telecare, so the literature on telecare was searched to understand how it is used by older adults to achieve goals of independence. Studies of any design and grey literature were included in the search. Pawson et al., (2005) describes this process as concept mining to determine key concepts, terms, and ideas for development and testing.

Enabling safety and facilitating ageing in place are key features of telecare recognised in the literature as important for supporting health and well-being [36]. Other key concepts included the use of telecare in promoting autonomy and fitting into a person’s everyday life [37]. The lead researcher (LF) used findings from the literature to develop IPTs in the form of if-then statements. These IPTs were then grouped into three overarching concepts—1) safety at home, 2) autonomy and choice, and 3) the integration of telecare into everyday life—which were used to develop a theoretically based evaluative framework for data extraction [31].

#### Step 2: searching for evidence

Formal literature searches in five databases were conducted in August 2023 (Medline, PsychINFO, Academic Search Ultimate, Web of Science, and CINAHL). The search terms were formulated with the assistance of a university librarian, who created a search strategy for each database using a combination of keywords, subject headings, and MeSH terms (see Supplementary Table 1, Additional File 1). The search terms were grouped into three categories: telecare technology, older adults and independence outcomes. Search terms were combined with Boolean operators. For each database, the search terms were restricted to the title and abstract fields only. We sought evidence on the use of telecare from 2003 onwards, providing a 20-year period for our search. This timeframe was selected based on the continued use of pendant alarms, despite their classification as older technology. Given that pendant alarms remain in use today, publications from the 2000s discussing their application were considered pertinent to this review. Telecare definitions are fraught with contradictions, and outcomes associated with independence vary considerably; therefore, the search criteria were kept broad to ensure that all potentially relevant sources were identified. This involved the use of various key search terms, such as “telecare”, “telehealth”, and “assistive living technology”. The criteria for independence outcomes were kept due to varying definitions of independence. Some studies focus primarily on physical health, emphasising the ability to function without assistance or reliance on others [38, 39]. However, other studies highlight broader meanings of independence which encompass maintaining a sense of control, self-esteem, self-determination, personal growth [39] and having access to resources to facilitate independence [40, 41]. As a result, a range of outcomes that are related to independence were included, such as physical health indicators (e.g. hospital admissions), mental health factors (e.g. anxiety/depression), loneliness and social isolation, quality of life, general well-being, autonomy, and resilience. A title and abstract screen, followed by a full-text screen, was subsequently conducted against the inclusion and exclusion criteria (Table 2). Citation details were stored and managed using Rayyan. The NIHR Public Advisors (JH and SA) conducted a random 10% check on the title and abstract and the full text to ensure consistency. Any discrepancies were resolved through discussions between the lead researcher (LF) and the National Institutes of Health (NIHR) Public Advisors (JH and SA). Forward and backwards citation tracking was utilised to reduce the risk of missing a significant document [43].

**Table 2:** Formal literature search inclusion and exclusion criteria

Intervention	Telecare interventions, referring to emergency help systems and fall detection systems.
Population	People described as older adults who live in their own home/community-dwelling. An age limit was not placed on the definition of an older adult, given the variety of ages that can considered as ‘older’ populations [42]; Older adults who receive formal or informal care.
Document type	Qualitative, quantitative, reviews, mixed methods research, or grey literature.
Outcome	Physical health (hospital admissions), mental health (anxiety/depression), loneliness and social isolation, quality of life, general well-being, autonomy, resilience.
Exclusions	Studies not written in English will not be included due to lack of resources required to translate studies. Interventions that do **not** include components such as emergency help systems or fall detection systems. Interventions that focus on monitoring vital signs (such as blood pressure) and report information back to a healthcare professional. Studies which focus on specific illness diagnoses (e.g. diabetes, dementia, chronic obstructive pulmonary disease (COPD)). Article corrections or retractions, book reviews, and abstracts that only reference talks.

#### Step 3: selection of articles

Thirty-seven papers were included at the appraisal stage. Traditional systematic reviews appraise the methodological quality of primary studies, usually through appraisal checklists. However, realist reviews also utilise emerging data across different document types that contain relevant data for theory development, refinement and testing [44]. Pawson (2007) argues that methodologically poor research can yield useful detail for developing theory [45]. Although there is no universal method for appraising documents for a realist review, Pawson et al. (2005) suggests assessing the ‘*relevance*’ of the information, which can be defined as whether the data contribute to theory building or testing, and the ‘*rigour*’ of the information, which refers to whether a piece of data is credible and trustworthy [46], by taking into account the methodology used.

To evaluate rigour and relevance, we adopted a broad assessment approach based on Williams et al., (2016) work, where the inclusion criterion was whether the evidence was ‘good enough and relevant’ [47]. For rigour, we assessed whether the authors clearly explained how the data were collected and whether the methods used were consistent with the results and conclusions. For relevance, we considered whether the evidence offered valuable insights for refining and developing the CMOs.

Papers were assessed for inclusion by scoring each document on its relevance and rigour [46] using a scale of high, medium or low to highlight lower quality studies and explore whether further evidence was required to support data that were low in terms of trustworthiness [46]. Member checking of the inclusion process took place within the research team. Papers deemed irrelevant were excluded (*n* = 5) because they did not contribute any data or evidence to the CMOs.

#### Step 4: data extraction

Data extraction was carried out on 32 papers using the theoretical framework template developed specifically for this review. A separate Excel spreadsheet was used to detail the study characteristics with full citation details, study design, data collection methods, results and relevance and rigour scores (see Supplementary Table 2, Additional File 2). Data that contributed to theory development and refinement were extracted (see Supplementary Table 3, Additional File 3). A section for notes was used in the template to record specific contexts, reported and perceived outcomes, and potential mechanisms to identify demi-regularities (patterns of mechanisms) and to facilitate further theory refinement and development of new theories at the data extraction phase [26].

#### Step 5: data synthesis

Data analysis was undertaken by the lead researcher (LF) with concepts and theories discussed with the wider research team (CH, YL and NH) and the National Institute of Health (NIHR) Public Advisors (JH and SA) in an attempt to ensure the credibility and trustworthiness of the inferences made. The data were read and reread for familiarisation, and patterns of contexts, mechanisms and outcomes were explored by iterative coding and grouping. Both inductive and deductive logic were used to form initial ideas and theories about what underlying powers might be producing the observed patterns in the data. An additional domain called ‘connection to social resources’ was added to the theoretical framework, as this was a reoccurring theme. The initial CMOs were created, and an iterative process of revising and refining the CMOs then occurred. The lead researcher (LF) conducted realist review training for the NIHR Public Advisors, and following this, refinements and additions were made through discussions with the Public Advisors in two 2-hour long meetings. Subsequently, the CMOs were further revised by returning to the data and actively extracting additional relevant information. The mechanism in the CMO configurations was presented in two parts—mechanism *resources* (what is offered by the telecare intervention) and mechanism *response* (how older adults respond to telecare resources)—to further explore the generative causation of *how* telecare works [48]. Additionally, the lead researcher drew upon middle-range theories (a developed theory that can be used to explain the cause of outcomes from an intervention) [26] to provide more theoretically informed explanations of mechanisms. Final discussions took place with the wider research team to finalise the CMOs.

## Results

### Study characteristics

**Fig. 1:**
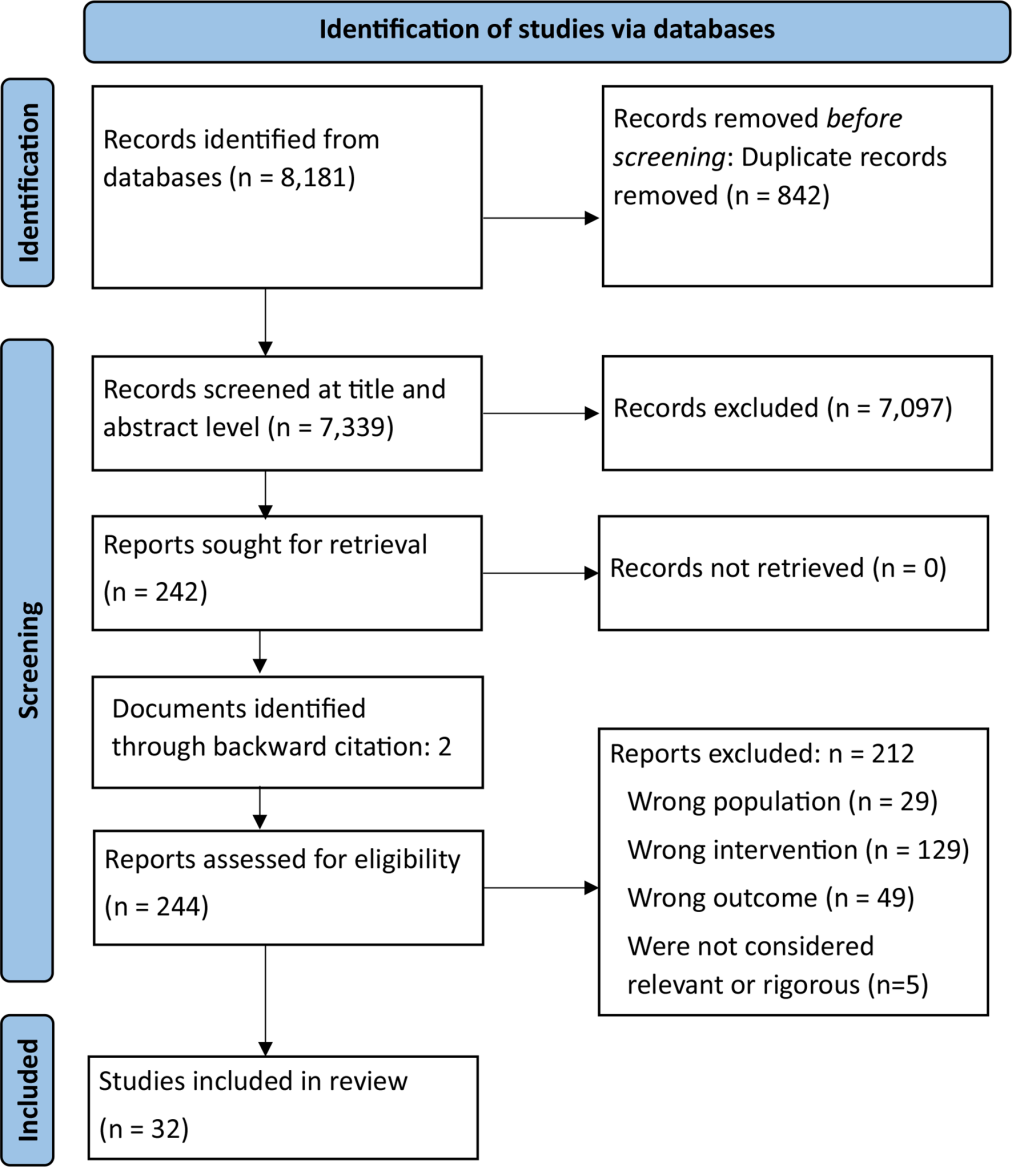
PRISMA flowchart of included articles [73]

Thirty-two studies were included in the review, as these documents contained relevant data needed to develop and refine theories. Figure 1 depicts the PRISMA flow diagram. The majority of the sources were published in the UK (*n* = 13). The remaining sources collected data from the United States of America (USA) (*n* = 4), Australia (*n* = 4), Canada (*n* = 1), New Zealand (*n* = 1), Hong Kong (*n* = 1), and six European countries (the Netherlands (*n* = 1), Finland (*n* = 1), Norway (*n* = 2), Spain (*n* = 2), France (*n* = 1), and Switzerland (*n* = 1)).

The findings are organised across four domains that explain how telecare might work and for whom. Twelve CMOs were constructed from the included studies. Table 3 provides a summary of the 12 CMOs, which are structured into four domains: (1) security at home, (2) autonomy and choice, (3) connection to social resources, and (4) integration into everyday life. A narrative summary of each CMO is presented.

**Table 3:** Summary of the 12 CMO configurations

CMO	Context	Mechanism	Outcome
Security at home			
CMO1: Connection to help.	Perceived risk of losing independence. Feeling vulnerable. Concern from family.	Having telecare that offers a connection to reliable help in an emergency (mechanism - resource) will allow the user to gain trust and faith in the technology to keep them living at home for longer, which will provide peace of mind and reassurance (mechanism – response) that they will receive the help they need.	Reduced anxiety related to risk management at home. Increased confidence to live at home.
CMO2: Ensuring privacy.	Perceived poor health/high risk of losing independence.	Passive monitoring which provides 24-hour monitoring (mechanism-resource) may provide additional support and peace of mind and reassurance to people willing to give up some privacy and control for additional safety support (mechanism-response).	Increased confidence in detecting risk/emergencies.
CMO3: Detecting subtle changes in health.	Conscious about declining health.	If telecare detects health and cognitive decline through data collection and informing users/carers of potential decline (mechanism-resource), this may provide opportunity to users to change their lifestyle or intervene at an earlier stage (mechanism-response).	Increased sense of active ageing. Likely to enhance ability of individuals to avoid disease/frailty.
CMO4: Meeting future needs.	People who want to plan for the future.	Telecare that is installed to meet *anticipated* future needs (mechanism-resource) may help older adults feel reassured that they have resources already in place (mechanism-response).	Preparedness for ageing. Reassurance of support Proactive support.
2) Autonomy and choice			
CMO5: Assessing needs.	Perceived risk of losing independence. Access to social services for assessment.	Conversations with telecare provider to assess individual needs and customise/match the telecare device to the individual needs (mechanism-resource) may increase sense of self-care (mechanism-response).	Increased control over perceived health-risk.
CMO6: Choice in using telecare.	Feels open towards using telecare.	Giving users the choice to use telecare and what kind of device they can use (mechanism – resource) may help the person feel empowered to self-govern oneself (mechanism-response).	Increased confidence to make decisions about own independence.
CMO7: Choice in *how* telecare is used.	Fear of being framed as frail or vulnerable.	Telecare that encourages control and choice in how it is used before and after an incident (mechanism-resource) may reduce feelings of being “burdensome” to those who provided support, and reduce the perceived image of being frail and needing support (mechanism-response).	Sense of control. Supporting personal and social identities.
3) Feeling connected to social resources			
CMO8: Providing social connections.	Limited social resources.	Having telecare that provides continual reassurance of connection to a wider system of support (mechanism – resource) may increase feelings of connectedness (mechanism – response). Having access to a social network that is accessible, friendly and welcoming (mechanism – resource) may help empower individuals to ask for help and use the network for social interaction (mechanism – response).	Reduced loneliness and social isolation.
4) Integration into everyday life			
CMO9: Understanding telecare.	Anxious about losing independence.	Ensuring understanding of how telecare works, how it can support independence and what will happen in the event of an alarm being raised (mechanism – resource) will enable trust in telecare to support independence and peace of mind (mechanism – response).	Reduce anxiety around losing independence/having a fall.
CMO10: Customising telecare.	Individual expectations and needs.	If the telecare technology can be customised and personalised to suit individuals need and preferences (mechanism-resource), then it will be more appropriate to a wider population with differing needs (mechanism-response).	Improved integration into everyday life. Improved ease of use.
CMO11: Familiar design.	Anxiety related to technology.	If the telecare technology has a design that the user is aware of and used to using (mechanism-resource), then the user will feel more confident that they can use it and help integrate better into daily routine (mechanism-response).	Improved efficiency in use.
CMO12: User expectations.	Older adult wishes to use telecare to achieve a specific goal.	When telecare matches user’s expectations (mechanism-resource), this will increase trust in technology to support the user to live independently (mechanism-response).	Increased feeling of safety. Increased confidence to live at home.

**Fig. 2:**
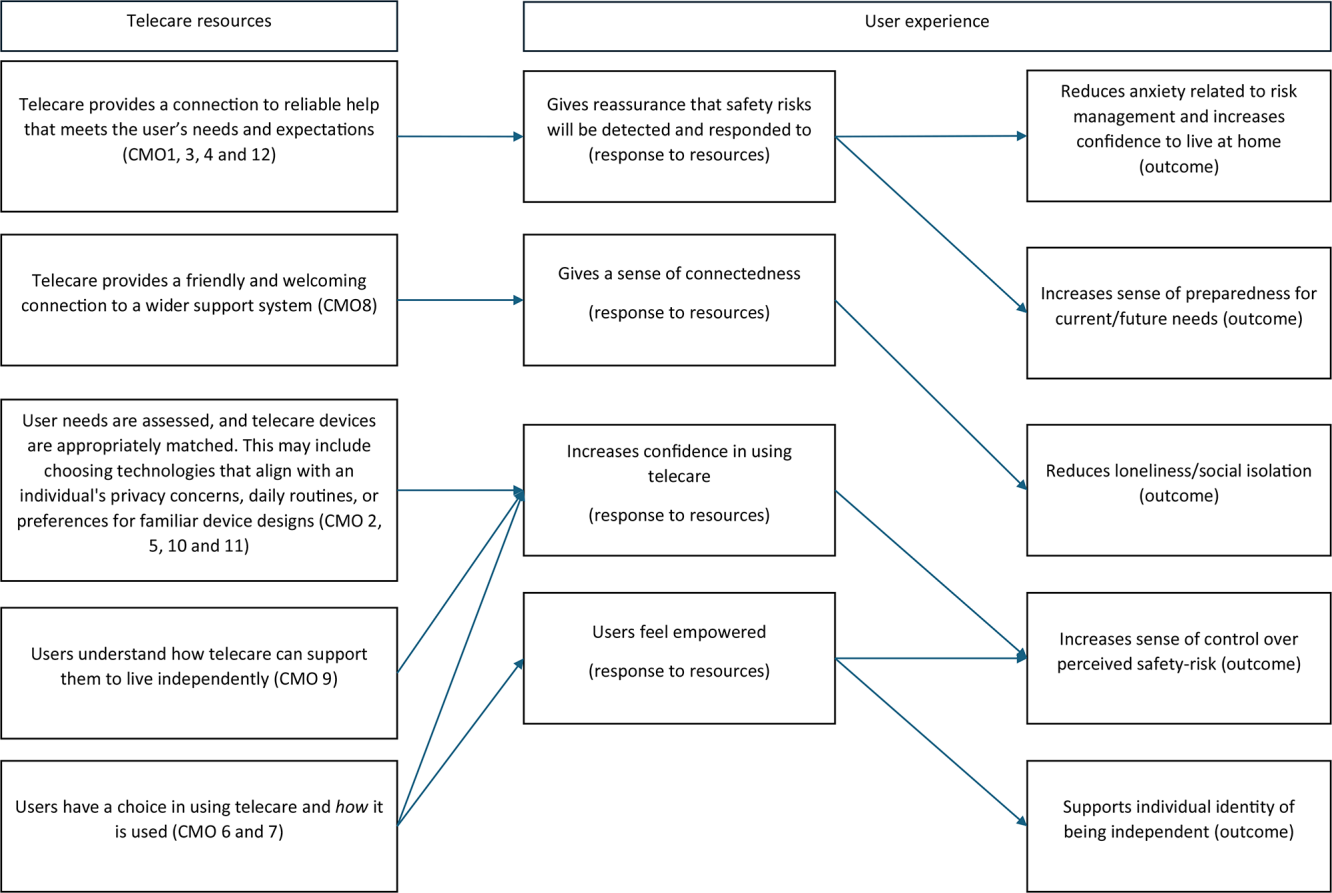
Overarching programme theory on how telecare can support independence in older adults. The programme theory demonstrates how the telecare resources may influence the user’s experience, which includes the individual’s response to these resources and the potential outcomes related to independence

In this realist review, the CMOs represent individual programme theories. We also present an overarching programme theory that integrates these individual CMOs across the four domains, aiming to highlight the key mechanisms that explain how telecare might work. This overarching theory was developed by examining the 12 CMOs and identifying shared mechanisms of importance (Fig. 2). These mechanisms are represented by telecare resources and the responses of older adults to these resources. The telecare resources were mapped to the responses of older adults, along with the potential positive outcomes that could occur when these mechanisms are activated. Figure 2 illustrates the overarching programme theory, highlighting the key mechanisms that explain how telecare may work. For example, if telecare provides a reliable connection to help that meets users’ expectations, it may offer reassurance that safety risks will be detected and addressed. This, in turn, could reduce anxiety about risk management, boost confidence in living at home, and increase preparedness for current and future needs. Another example from Fig. 2 shows that if a telecare device is well-matched to the user’s needs, such as using a device with a familiar design or one that fits into their daily routine, the user is likely to feel more confident using the technology, which in turn enhances their sense of control. Figure 2 illustrates how each individual CMO contributes to the overall programme theory, by labelling which CMOs are associated with each telecare resource presented in the programme theory.

#### Domain 1: security at home

##### CMO1: providing a connection to help

Our analysis highlighted that older adults overwhelmingly wished to use telecare to feel safe, to remain living in their own homes [18, 49–56], and to delay transfer to institutional care [49, 51, 57]. Most older adults recognised the increased risk of injury following a fall if care was delayed. Therefore, users wished to be connected to a source of help in an emergency [7, 54, 57–64], which was a key factor for telecare use and acceptance [54, 65, 66]. The need to be connected to a source of help is mostly aligned with a perceived risk of losing one’s independence [49, 67]. The reliability of telecare was seen as an important mechanism to ensure that help could be sorted quickly [49, 64, 68–71]. Our findings also highlighted that trust in telecare was crucial for reducing anxieties related to having a fall [66, 70, 72, 73]. Previous negative experiences with telecare, where help was not delivered in a timely manner, led to distrust in telecare services, which increased the risk of discontinued use or increased anxiety in anticipation of a critical event [72].

##### CMO2: ensuring privacy

Our findings revealed that older adults with a high perceived risk of losing their independence were more willing to relinquish some control over their lives in return for feeling safe. These users were more willing to use more ‘invasive’ monitoring telecare services, such as passive and ambient sensors [36, 67, 74–77], whereas for others who did not perceive a high risk of losing independence, passive monitoring was more likely to impact their identity [78], autonomy, and self-efficacy to live independently [57]. This finding highlights the importance of involving users in the assessment of the appropriateness of telecare. Understanding what data are collected from devices, who views them and how they are used is important for increasing trust in telecare and reducing fear [76, 77, 79]. Ensuring that older adults understand how data are used and giving individuals the choice over what data are shared with others may reduce anxiety related to privacy.

##### CMO3: detecting subtle changes in health

A few studies have discussed the perceived value of telecare in detecting health deterioration. Older adults appreciated that monitoring technologies could detect changes in behaviour that may have gone unnoticed by themselves or others around them [55, 56, 67, 80]. Cognitive decline was of particular interest, particularly for those who had witnessed the perceived undesirable impacts of dementia on relatives or friends [67]. However, not all older adults held this interest, and some were resistant to the notion of detecting subtle changes in their daily behaviour patterns, as it was viewed as invasive [67, 68]. For some, detecting early deterioration, particularly cognitive decline, was associated with negative stereotypes of dementia and memory loss, which was viewed as a threat to their identity and the future of their independence [56].

##### CMO4: meeting future needs

Our findings revealed that some older adults used telecare in anticipation of meeting future security needs that they were anticipating [61, 72, 81]. Having telecare resources already available in their home environment provided reassurance of security and was viewed as enhancing the sustainability of living in their own home [65]. This CMO refers to our initial theoretical foundation, where older adults acquire new technological resources to achieve their goal of remaining at home [34].

#### Domain 2. autonomy and choice

##### CMO5: assessing needs

Our findings highlight that telecare is often offered as a standard package without fully understanding the preferences of older adults [10, 82], which often results in non-use, discontinuation, or adaptive use that supports users’ priorities, such as control, autonomy, privacy, safety and connection to social resources [10, 54, 57, 65, 83, 84]. Predefining users’ needs and ignoring the individual context may elicit a sense of not being heard. Our findings emphasise the importance of understanding users’ needs and preferences, matching the type of telecare, and informing decisions about how their support network is involved [10, 36, 64]. Greenhalgh et al., (2013) reported in their ethnographic study that some older adults may have a poor understanding of how to access help, compounded by other inequalities such as low technological confidence and low social support to support telecare use. If telecare is not aligned with an individual’s needs and preferences, it can lead to unintended consequences, such as unmet personal safety concerns [83] or a sense of intrusion into their autonomy and privacy [10, 36, 82, 84, 85]. This is especially true when the individual does not perceive a need for telecare, which is often the case with passive monitoring [57]. Older adults have different illness experiences and varying levels of social and financial resources [83]; hence, a standardised approach to telecare implementation may not be appropriate.

##### CMO6: choice of telecare

Studies revealed that older adults who actively chose to use telecare felt more in control and more empowered to use the device [18, 72, 82, 86]. The reasons for choosing telecare mostly stemmed from the desire to remain at home. Some older adults felt that this was a decision that had to be made to stay living at home and accepted that some control would have to be traded to accomplish this goal [72, 76, 83], while others felt pressure from social services and relatives to take on telecare [18]. Feeling pressured to use telecare enforced a perceived identity of becoming frail and constantly at risk of decline [18]. Therefore, enabling choice is a key mechanism that may elicit a feeling of control [80], empowering people to maintain behaviours such as utilising telecare, which will allow them to stay at home longer [82].

##### CMO7: choice in how telecare is used

The choice of how to use telecare was a salient theme for some older adults. Findings suggest that some older people may be hesitant to share their daily behaviours or publicise difficulties by engaging with telecare [54, 74, 84]. While these individuals still seek reassurance about safety, as noted in CMOs 1 and 2, those concerned about being perceived as frail may prefer greater control over *how* the telecare device is activated. For example, older adults who are fearful about appearing frail may prefer to decide when the telecare system is triggered to request assistance. Brownsell & Hawley, (2004) found that older adults do not always want falls to be known about due to fear of being pressured to move into institutional care or being hospitalised. Older adults may also be concerned with maintaining the social identity of being independent and wanting to avoid the stigma of identifying as frail or dependent [54, 68]. Giving older adults who wish to avoid being seen as vulnerable control over how telecare is used is important for maintaining their self-esteem and preserving their sense of independence [80]. In contrast to this, CMO2 suggests that older adults who are less concerned about being perceived as frail may prioritise safety over control and feel comfortable being monitored to ensure more reliable assistance in an emergency. Balancing the desire for control with the need for safety can be challenging, and some older adults may be reluctant to give up any control, even for the sake of safety.

#### Domain 3: feeling connected to social resources

##### CMO8: providing social connections

The older adults in these studies highlighted the importance of maintaining and securing connections to social resources through the use of telecare. This manifests in the fear that telecare may increase social isolation through decreased face-to-face interaction [51, 64, 70, 77, 84]. Felber et al. (2023) reported that telecare could not replace human connection, as it could not provide the same level of relationship, which is possible with face-to-face communication. Those who use telecare may be at risk of social isolation, particularly if they are housebound or live alone, and as a consequence, telecare could provide an avenue for social connection [61]. Notably, Percival & Hanson (2016) reported that older adults pressed their alarm button purely for human interaction rather than using it for its ‘designed’ purpose to help in emergencies [82]. In this example, telecare could provide an avenue for social support; however, the need for social connection should be recognised as a legitimate use of telecare. Indeed, a level of empathy and care is needed by telecare providers to empower users to reach out for emotional support and to help build strong relationships [52].

#### Domain 4: integration of telecare into everyday life

##### CMO9: understanding telecare

The findings revealed that older adults wanted to understand exactly how telecare worked and what was required of them to cope and manage in an emergency event [62, 70, 72]. Research has identified challenges for older adults in understanding how telecare works; for example, if the person has cognitive or memory impairments, they may not easily retain information [70]. Other instances may include uncertainty around the workings of telecare and the processes of what happens when an alarm is triggered, i.e., who will come to help and how will they access the property [62]. When older adults lack an understanding of how telecare works and are anxious about losing their independence, this may result in feelings of uncertainty around the perceived reliability of telecare [72], reducing the likelihood of gaining reassurance of safety [62].

##### CMO10: customising telecare

Telecare devices were described as an extension of that person and their values and thus needed to fit into the user’s current life. The importance of assessing the needs of the user to implement appropriate telecare devices has already been covered. However, individual needs and preferences may change over time, and if the technology no longer ‘works’ in this new context, it may lose its value and interfere with the individual’s life, leading to disengagement and non-use [50, 78]. It was highlighted that telecare devices that involved pragmatic customisation in which devices were adapted and combined with existing technologies already in the home were better suited to individuals’ needs and preferences [68]. Being able to customise telecare may help to align the technology with the individual’s self-perception and identity [84]; for example, the functionality of setting reminders may work for someone who experiences forgetfulness [54]. Ensuring that the telecare device matched the person’s identity was found to be critical to the adoption of telecare and continued use [18, 71, 76, 82, 84, 87].

##### CMO11: familiar design

Positive experiences were elicited when telecare did not interfere with the person’s daily routine, and the technologies were not noticeable in their home environment [63, 84]. For older adults who experience anxiety towards new technologies, having a technology that has a familiar design to that individual may reduce anxiety related to technology and facilitate better ‘fit’ into that person’s environment, for example, implementing a tablet-style device for someone who is familiar with using tablets [88].

##### CMO12: user expectations

Our findings highlighted the need to meet user expectations to ensure continued use and benefits to well-being. When telecare devices were inaccurate, for example, when sensors were too sensitive or not sensitive enough, older adults stopped using them [52]. User expectations of what telecare should provide may differ, as some older adults may wish to have a quick response time in an emergency, while others may prioritise reducing false alarms [62, 63, 72]. Providing information on the efficiency and effectiveness of telecare devices to users may enable individuals to choose devices that meet their expectations [52].

## Discussion

This realist review explored how telecare can support independence in older adults. It contributes a programme theory that highlights key mechanisms for how telecare may support different preferences and needs, which may be useful in improving the uptake and use of telecare.

A key independence goal for older adults was to remain at home, and feeling secure at home was critical. Telecare that provided a connection to help, meeting an individual’s expectations of the device, led to reassurance of safety and reduced anxiety. Reasons for using telecare to improve safety at home differed across individuals, as some recognised their personal need for the device [10] due to poor health, while others wanted to feel prepared for future anticipated needs [65]. Some older adults wanted to use telecare to detect potential declines in their health to ease anxiety [55, 56]. The overarching programme theory (Fig. 2) highlights the importance of assessing individual needs when matching telecare devices to individuals. These findings align with the SOC model presented at the outset of this research [32] in regard to how older adults select goals based on preferences, personal motivation, and age-related losses and adapt accordingly to achieve these goals, which in turn improves quality of life and well-being. Despite this, research involving an online survey of English local authorities revealed that telecare was often provided without prior assessment of the person’s preferences or needs [23]. Telecare tends to be installed very quickly after an emergency or following hospital discharge, which creates challenges in conducting initial assessments [89].

Having personal choice in using telecare facilitated personal decision making and autonomy, a crucial component of programme theory. An important context for choosing to use telecare was recognising their own risk of losing independence. Studies have demonstrated reluctance from older adults to adopt telecare devices, as it can often be associated with perceived stigma [19, 90]. When older adults had a perceived risk of losing their independence, using telecare led to positive outcomes, including peace of mind, reduced anxiety, and increased control over the perceived health risk [82, 86, 91]. On the other hand, if older adults had a fear of being framed as frail or did not perceive themselves as at risk of losing their independence, the mechanism of feeling forced to adopt telecare led to feelings of being stigmatised and impacted self-identity [65, 82]. Research has reported negative impacts resulting from self-perceived ageism and revealed associations between negative attitudes towards ageing and poorer mental health, such as depression [92]. Other studies suggest that self-perceived stigma is associated with widespread negative consequences, including lower quality of life, premature mortality, and poorer physical health [93].

Ensuring that telecare is integrated into a person’s life was another key area highlighted in the programme theory. A barrier to use was a lack of understanding from the older adult’s perspective of how telecare worked and how it would support independence. Ensuring understanding may require different approaches, given the varied contexts among older adults who adopt telecare, including anxiety towards new technologies, existing cognitive impairments or individuals experiencing personal issues, that led to the need for telecare (hospitalisation, illness, bereavement). This finding relates to the ecology of ageing, which posits that in order for environments to enhance opportunities for ageing well, environmental resources such as telecare should match personal competence [94]. Telecare staff should adapt their communication style accordingly when implementing telecare to ensure that older adults understand how it works. To help match technologies to individuals and their personal context, telecare staff should also be aware of personalised solutions, for example, by giving an older adult who may be anxious about using new technologies a device that has a design that is familiar to the device recipient. However, recent literature has highlighted issues with telecare staff training, which may impact staff knowledge. Woolham et al. (2019) reported varied levels of knowledge and awareness about telecare among staff [23]. Greenhalgh et al. (2015) found that social workers and care managers saw the need for personalised technological solutions but lacked the means to deliver them [24]. Our findings suggest that the impact of telecare on supporting independence is influenced by user understanding of telecare and the extent to which technology is matched to the individual. However, this is highly dependent on the telecare staff’s knowledge and awareness of telecare and the useability of the device. Further research should investigate how to improve and standardise telecare training to ensure the integration of telecare into the recipient’s environment.

The overarching programme theory highlighted the potential for telecare devices to provide social connections to older adults with limited social networks. Older adults may benefit from additional sources of social connection, as within their context, older adults are more likely to have limited mobility and may not be able to form and maintain new contacts outside the house [95]. Telecare offers an accessible route to social interactions, as most telecare devices do not require the internet and are based in the home environment. However, within social care, telecare is not currently used to supply social support. The utilisation and feasibility of telecare in providing this service may require further research.

### Limitations

Despite the inclusive search strategy, people from minority ethnic backgrounds remain underrepresented in this study. This is partly due to the lack of literature focused on the experiences of minority ethnic groups using telecare. This impacted our ability to fully explore ‘for whom’ telecare works. We suspect that telecare may not work for every cultural group, as individuals may have vastly different contexts in which telecare is situated. However, the lack of research in this area prevents researchers from testing hypotheses. Following this review, new research may be designed to address this gap in knowledge. Although a few studies included longitudinal data, most studies did not evaluate older adults’ use of telecare and changing needs over time. Further research is required to understand how to retain the usefulness of telecare for long-term use.

## Conclusions

Our findings in this realist review highlight the importance of understanding not only the physical needs but also the psychological and social needs of older adults to be able to implement telecare impactfully. Telecare assessments should be conducted to support autonomy by enabling choices of technological resources, including the level of monitoring, freedom to call for help if needed, links to social support, and ability to customise technology to suit needs. Telecare devices should support older adults’ goals of staying at home and feeling secure, which may differ among individuals. To support this, telecare should provide reassurance of help in an emergency, enable connections to existing/new social networks, and help individuals detect age-related deterioration to prevent further loss. Finally, telecare must integrate into everyday life by fitting people’s existing environment, skills, capacity, and identity. A realist approach enabled us to unpack hidden mechanisms that may enable social care professionals to tailor their approach to implementing and utilising telecare to support older adults.

## Electronic supplementary material

Below is the link to the electronic supplementary material.

Supplementary Material 1

Supplementary Material 2

Supplementary Material 3

## Data Availability

The datasets used and/or analysed during the current study are available from the corresponding author upon reasonable request.

## Abbreviations

ADL: Activities of daily living
CMO: Context-Mechanism-Outcome
IPT: Initial programme theory
MRT: Middle Range Theory
NHS: National Health Service
NIHR: National Institute of Health and Care Research
RCT: Randomised controlled trial
SOC: Selective Optimisation with Compensation
UK: United Kingdom
