# Supplementary material for: Understanding how, for whom and under what circumstances telecare can support independence in community-dwelling older adults: a realist review
Source: BMC Geriatr. 2025 Jan 27;25:59. doi: 10.1186/s12877-024-05650-6 (PMC11771067; doi:10.1186/s12877-024-05650-6)
Supplement: Supplementary file 1 — Supplementary Material 1 [file 12877_2024_5650_MOESM1_ESM.docx]

Additional file 1. Search terms

For each database, the search terms were restricted to the title and abstract fields only. For the papers and articles identified in the search, titles and abstracts were screened first, followed by a full-text review, with both steps applying the inclusion and exclusion criteria.

| **Database** | **Search terms** |
| --- | --- |
| **Medline** | telecare OR telehealth OR assistive living technology OR wear* device* OR smart home technology  AND  psychological well-being OR well-being OR psychological wellbeing OR wellness OR quality of life OR anxiety OR depression OR mental health OR control OR self-regulation OR loneliness OR stress OR emotional OR autonomy OR positive mood OR wellness  AND  independent living OR community-dwelling older adults OR older adults OR elder* OR seniors OR frail elder*  NOT  Dementia  Child*  Care homes |
| **PsycINFO** | telecare OR telehealth OR assistive living technology OR wear* device* OR OR smart home technology  AND  psychological well-being OR mental health OR control OR anxiety OR depress* OR agency OR self-regulation OR emotional control OR quality of life OR loneliness OR stress OR emotional OR autonomy OR positive mood  AND  independent living OR community-dwelling older adults OR elderly OR seniors OR older adults  AND NOT dementia AND cancer  Children  Care homes |
| **Academic search ultimate** | telecare OR telehealth OR assistive living technology OR wear* device* OR smart home technology  AND  psychological well-being OR well-being OR anxiety OR depression OR mental health OR control OR agency OR resilience OR self-regulation OR loneliness OR stress OR emotional OR autonomy OR positive mood  AND  older adults OR independent living |
| **Web of Science** | telecare OR telehealth OR assistive living technology OR wear* device* OR OR smart home technology  AND  Older adults OR independent living OR elder  AND  Psychological OR wellbeing OR anxiety OR control OR agency OR self-regulation OR mental health OR quality of life OR depression OR resilience |
| **CINAHL** | telecare OR telehealth OR assistive living technology OR assistive technology OR wear* device* OR smart home tech*  AND  psychological well-being OR well-being OR wellbeing OR psychological wellbeing OR wellness OR quality of life OR anxiety OR depress* OR mental health OR control OR self-regulation OR loneliness OR stress OR emotional OR autonomy OR positive mood OR wellness OR psychological resilience OR psychological frailty  AND  independent living OR community-dwelling older adult* OR older adult* OR elder* OR senior* OR frail elder* |
