# Supplementary material for: Understanding how, for whom and under what circumstances telecare can support independence in community-dwelling older adults: a realist review
Source: BMC Geriatr. 2025 Jan 27;25:59. doi: 10.1186/s12877-024-05650-6 (PMC11771067; doi:10.1186/s12877-024-05650-6)
Supplement: Supplementary file 2 — Supplementary Material 2 [file 12877_2024_5650_MOESM2_ESM.docx]

Additional file 2. Study characteristics of included studies

| Author and year | Study methodology | Study focus | Study setting and sample | Country | Intervention | R&R |
| --- | --- | --- | --- | --- | --- | --- |
| Lynch et al. 2022 | Qualitative interviews and ethnographic methods | Aimed to explore how reassurance emerges through human-technology interactions? | Older adult users of pendant alarms. Age not specified. N=19 | United Kingdom | Pendant alarm | High usefulness |
| Mortenson et al. 2016 | Qualitative semi-structured interviews | Aimed to explore how surveillance technologies change the way older people experience the home environment? | Older adults aged 60 or over. Presented an ambient assistive living technology during the interview. N=27 | North America (Canada and US - Vancouver, British Columbia and San Francisco, California) | Ambient assistive living presented to participants in a video | High usefulness |
| De San Miguel and Lewin 2008 | Retrospective quantitative survey with free text comments. | Aimed to determine if people used their alarms, and to explore how this type of technology impacts on older individuals lives and why. | Older adults who had used pendant alarms for 6 months or longer. 75% of users were 80 years or older. N=1476 | Australia | Pendant alarm | Moderate usefulness |
| Berridge  2017 | Qualitative semi-structured interviews | Aimed to explore how older people adopt, refuse, discontinue and adapt to using passive monitoring systems. | Long term telecare users (6 years). Older adults aged 65 and over. N= 15. | United States of America | Five sensors installed in specific predefined locations within apartments. Telecare service provided through a call centre. | High usefulness |
| Chaudhuri et al. 2017 | Qualitative focus groups | Aimed to explore the experiences and perceptions of older people on fall detection technologies and their willingness to use such devices. | Older adults aged 60 and over, living in independent and assisted living communities. Explained a theoretical telecare device and asked opinions on it. N= 27. | United States of America | Fall detector device – call button and ambient sensors to detect a fall. | High usefulness |
| Johnston et al. 2010 | Qualitative semi-structured interviews | Aimed to explore the experiences of older people who had fallen recently and their thoughts on pendant alarms. | Older adults aged 65 years and older who had sustained a fall in the previous six months. N=31. 20 participants currently used pendant alarms. | Australia | Pendant alarm | High usefulness |
| Brownsell and Hawley 2004 | Quantitative Pre and post intervention study with control group. Qualitative interviews afterwards. | Aimed to explore the impact of telecare on reducing the fear of falling. | Older adults currently using automatic fall detectors aged over 75 years or those aged 60–74 years who had experienced a fall in the previous six months. Monitored over 17 weeks. Assigned to a control group (n = 21) or intervention group (n = 34). N=55. | United Kingdom | Automatic fall detector | Moderate usefulness |
| Elers et al. 2018 | Qualitative semi-structured interviews | Aimed to explore the experiences of telecare use in older adults | Older adults aged 74-92 years. Participants were asked about their needs and how telecare could fulfil their needs. N=10. | New Zealand | Home monitoring technology. | High usefulness |
| Hamblin 2016 | Qualitative, multi-method, longitudinal research study - ethnographic observations, qualitative interviews and photography and diaries. | Aimed to understand factors which influence telecare acceptance and usage. | Older adults aged 65 and over. Participants were new telecare users, or had been telecare users for 12 months. N=60. | United Kingdom | Pendant alarm | Moderate usefulness |
| Greenhalgh et al. 2013 | Ethnographic methods. | Aimed to develop a theoretical model of assistive technology use | Older adults aged 60 and over. N=40. Participants were visited at home and asked about their use (or non-use) of technologies. | United Kingdom | Assistive living technology – including telecare in the form of pendant alarms | High usefulness |
| Brownsell et al. 2008 | Quantitative controlled trial | Aimed to quantify the impact of telecare on users, specifically to understand the impact  on people’s health and wellbeing | Older adults aged over 70. 24 participants were in the intervention group, with a control group of 28 people. N=52. 12 month monitoring period. | United Kingdom | Flood detectors and temperature  Detectors,  fall detectors and automatic light switch, movement detectors. | Moderate usefulness |
| Bowes and McColgan 2012 | Qualitative semi-structured interviews | Aimed to understand how far telecare can support older people’s goals of independence, participation, and identity. | Older adults aged 60 years and older receiving telecare services at home and in housing with care. N= 76. Study conducted over 3 years. | United Kingdom | Home alert system linked to a telephone, with two passive detectors, which can detect movement, flood detectors, heat sensor and smoke detector. | High usefulness |
| Wild et al. 2008 | Qualitative focus groups | Aimed to identify monitoring needs and expectations of community-residing elderly and their family members. | Older adults aged 65 years and older. Participants were asked about their opinions on a potential home monitoring sensor. N= 23. | United States of America | In-home monitoring – ambient sensor | Moderate usefulness |
| Watson et al. 2021 | Qualitative semi-structured interviews | Examined the impact on service users of rapid response services in social housing. | Participants included older adult service users and vulnerable service users. Age not specified. N=10 | United Kingdom | 24/7 emergency response service combined with a telecare service | Medium usefulness |
| Karlsen et al. 2017 | Qualitative systematic review | Aims to review qualitative evidence of community-dwelling older adults' experience with the use of telecare in home care services. | This review considered studies that examined older adults' experiences with the use of active and passive technology devices. Aged 60 and over. | Norway | Personal alarms and sensor technology | High usefulness |
| Percival and Hanson 2016 | Qualitative focus groups | Aimed to explore priorities of older people regarding possible uses of telecare services. | Carried out focus groups with 10 older people. Age not specified. Participants were presented with specially designed case scenarios related to telecare devices. N=10 | United Kingdom | Flood detector, fall detector, bed sensor | Moderate usefulness |
| Pol et al. 2016 | Qualitative semi-structured interviews | Aimed to determine the perspectives of older people regarding the use of sensor monitoring in their daily lives. | Older adults aged over 68 who had a sensor monitoring system installed in their home for 1 and a half years. N=11 | Netherlands | Sensor monitoring system – ambient sensors | High usefulness |
| Nyman and Victor 2014 | Quantitative cross-sectional survey | Aimed to investigate telecare use in older adults. | Users of personal call alarms among community-dwelling adults aged 65 and over. N= 3,091 | United Kingdom | Pendant alarm | High usefulness |
| Leikas and Kulju 2018 | Qualitative focus group | Aimed to understand ethical issues related to monitoring technology. | Older adults aged 70 years and above, using ambient sensors for study purposes. N=8. | Finland | Movement sensors (ambient) | High usefulness |
| Karlsen et al. 2019 | Qualitative semi-interviews | Aimed to understand use of telecare for older adults and their family caregivers. | Older adults aged 60 years and above and a received telecare service within the last 0–3 months. N=18. | Norway | Telecare – first to third generation. | High usefulness |
| Hamblin et al. 2017 | Ethnography – interviews and field data (mixed method) | Aimed to understand factors which affect the optimal use and implementation of telecare. | Older adults aged 65 and above who had been assessed as at risk of falls. N=60. | United Kingdom | Telecare – first to third generation. | High usefulness |
| Berridge et al. 2019 | Qualitative semi-interviews | Aimed to examine the experiences and insights of low-income, immigrant senior residents that offered a sensor-based passive monitoring system. | Older adults using a sensor-based passive monitoring system. Age not specified. N=20. | United States of America | Sensor-based passive monitoring system. | High usefulness |
| Aceros et al. 2015 | Ethnographic methods – interviews, focus groups (quali mixed method) | Aimed to explore what was meant by ‘good aging’ and how it is constituted in telecare practices. | Older adults who currently used a telecare service consisted of a personal alarm system. Age not specified. N=10. | Spain | Pendant alarm | Moderate usefulness |
| Pech et al. 2021 | Literature review | Aimed to review telecare devices to support older people. | Review of literature exploring telecare use in older adults, age not specified. | France | Telecare – first to third generation. | Low usefulness |
| De San Miguel et al. 2017 | Cohort study | Aimed to assess effectiveness of telecare in providing assistance in an emergency and other health and well-being outcomes. | Older adults aged 65 years or older who either used telecare or had expressed an interest in using it. N=295. Community-dwelling. | Australia | Pendant alarms | Moderate usefulness |
| Camp et al. 2022 | Semi-structured interviews | Aimed to understand older adults' opinions on the kid of telecare they would be willing to use within their own homes. | Older adults split into two groups: younger group (aged 55-69 years) and an older group (≥70 years). N=32. Community-dwelling. | United Kingdom | Ambient sensors | Moderate usefulness |
| López & Domènech 2008 | Ethnographic qualitative interviews | Aimed to understand how autonomy is embodied through the use of a telecare device. | Data extracted from 12-month ethnographic research study in a Catalan Telecare Service. Age not specified. Community-dwelling. | Spain | Telecare package – red button/pendant | Moderate usefulness |
| Pirzada et al. 2021 | Literature review | To understand more about the low acceptance and adoption of technologies among older adults. | Older adults using technologies related to smart homes to promote independence. Age not specified. Community-dwelling. | United Kingdom | Smart Home Technology | Moderate usefulness |
| Peng et al.2023 | Structured questionnaire survey | Aimed to investigate the factors associated with the telecare acceptance for older adults in Hong Kong. | Older adults aged 60 and above. N=110. Community-dwelling. | Hong Kong | Telecare – sensor technology | Moderate usefulness |
| Reyes et al.2023 | Semi-structured interviews | Aimed to understand the types of technologies that older adults use to support their health and motivations behind use. | Older adults aged 65 years and above. And used a device for their health care. N=22. Community-dwelling. | Australia | Telecare – sensor technology | Moderate usefulness |
| Felber et al.2023 | Systematic review | Aimed to investigate how ethical questions are discussed in the field of Smart home health technologies in caregiving for older persons. | Older adults aged 65 years and above and caregivers. Community-dwelling. | Switzerland | Telecare – sensor technology | Moderate usefulness |
| Fawcett and Karastoyanova 2023 | Semi-structured interviews | To analyse how Covid-19 affected older people in three areas of Scotland | Older adults in receipt of telecare services. Mean age was 74. N=29. Community-dwelling. | United Kingdom | Telecare – pendant alarms | High usefulness |
