# Supplementary material for: Understanding how, for whom and under what circumstances telecare can support independence in community-dwelling older adults: a realist review
Source: BMC Geriatr. 2025 Jan 27;25:59. doi: 10.1186/s12877-024-05650-6 (PMC11771067; doi:10.1186/s12877-024-05650-6)
Supplement: Supplementary file 3 — Supplementary Material 3 [file 12877_2024_5650_MOESM3_ESM.docx]

Additional file 3. Data extracts and notes from CMO development and refinement.

| CMO | Source | Data extract | Notes |
| --- | --- | --- | --- |
| CMO1: Providing a connection to help | Watson et al., 2021, page 900. | "*The first time [I fell] I was laid for hours before I managed to get to a phone to call help. I ended up at hospital and staying for 3 days. That’s why I signed up for rapid response. The last time was very different: I pressed the button and they were here in minutes, lifted me up and went on their way. Brilliant*." (SU09). | (pendant alarm) (housing association context) - rapid response to an emergency - Rapid response team providing care faster than NHS - fast response is crucial to peace of mind. |
| CMO2: Ensuring privacy | Mortenson et al., 2016, page 12. | “*As you get older…you lose a great deal of your privacy*” suggesting that AAL needed to be seen in the context of other personal care services and surveillance already in place. Similarly, many participants indicated they were not as concerned with AAL surveillance because they were already being monitored. "*Well, my neighbour across the road and I have this thing with the blinds, if my blind isn’t up by 10 o’ clock [in the morning], she will either phone or come over and see if I’m alright, we have each other’s keys, and she is a diabetic, so if she’s not up by 10 o’clock I check on her (Sarah).*" This example illustrates an informal, reciprocal kind of monitoring. | If monitoring technology is viewed as facilitating safety, users may accept foregoing privacy. Privacy seen as something lost in older age - acceptance of ageing. |
| CMO3: Detecting subtle changes in health | Pol et al., 2016, page 488. | Mr. A expressed: “*Look there are my sensors, they are my watchdogs, and they look after me*.” The sensors were able to capture things that the participants did not notice. Mr. A expressed this as follows: “*if there should be a slow change in my daily pattern, I certainly wouldn’t report it. I wouldn’t notice*.” | Monitoring helps to detect early indicators of health deterioration. Works towards staying at home – longer goal. Preventive tool. |
| CMO4: Meeting future needs | Hamblin et al., 2016, page 8. | Future needs had an inverse effect on obtrusiveness for some participants: rather than rejecting telecare because it may not meet future requirements, some had it installed to meet needs they were anticipating. | Some people were proactive rather than reactive. Preparedness is important to some. May not be relevant for all, for some perceived need is required. May be linked to stigma around technologies. |
| CMO5: Assessing needs | Lynch et al., 2022, page 6. | Gordon, an older man with multiple chronic health conditions, talked about the issuing of his community alarm device as indicative of the council’s consistent failure to meet his needs: Gordon: *“The council have always let me down in not getting the help that’s needed [...] What annoys me most of all, they put stuff on that I’m not really interested in, and they talk a lot of rubbish, a lot of them*.” | When technology does not match needs, frustration and feeling of being let down. Ignoring contexts results in user not attaching meaning to technology (pendant alarm). |
| CMO6: Choice in telecare | Hamblin et al., 2016, page 8. | *"I’m now beginning to understand, as age is catching up with me, that I am being forced – notice my word here – I am being forced in little ways to become dependent on somebody else. I’m being checked up on … it’s an interesting aspect but not one that I necessarily like. I’ve been too independent for too many years … it’s nice to know that they are there in the background but, how can I put this, I don’t want to become dependent" (Mr Eaves, 70s).* | Some feel forced to use telecare. Viewed as a lesser of two evils, struggle between wanting to stay at home and wanting to remain independent (telecare may impede independence if it's not seen as a true ‘choice’). |
| CMO7: Choice in how telecare is used | Percival and Hanson., 2016, pages 894-895. | Positive implications of telecare for individual choice and self-determination were also raised. Participants thought that ICT may provide an older, virtually housebound person such as Mr Agnew (case scenario two) with more choice in respect of the ways in which he interacts, to be ‘in control of his own world and his own contact’. Behind many of the comments raised in the context of choice was that it is closely aligned with older people’s sense of self-determination in running their own lives. | People having a choice in *how* telecare is used will enable better matching to individual needs. Gives a sense of empowerment through facilitating autonomy. |
| CMO8: Providing social connections | Percival and Hanson., 2016, page 900. | an alarm call centre manager, spoke of the frequency with which service users press their alarm button, purely to hear a human voice and have ‘a chat’. The manager, frustrated that he only has resources to deal with life and limb emergencies, is now of the opinion that ‘emotional support’ maybe a ‘legitimate’ use of the alarm service, which needs to be costed so that the potential for having necessary staff available is examined. | Shows individuals with limited social resources (contexts) seeing telecare as a route to social connection. |
| CMO9: Understanding telecare | Lynch et al., 2022, page 114553. | Harry did not understand the workings of the community alarm or why, when he pressed its button, someone would try to talk to him through the white box by his television. Rather than offering Harry a connection, the technology offered a hermeneutic relation which made no sense, provoking anxiety rather than reassurance. *"Harry: Making me wonder if they’re trying to get in touch for any reason. I: And do they try and call you through that [community alarm box]? Harry: I’m not sure."* | Understanding telecare important in reducing anxiety towards telecare, and important in providing reassurance. |
| CMO10: Customising telecare | Karlsen et al., 2019, page 1307. | “*When memory gets worse it is ok to receive reminders, because then you remember it, and then you can do it.”* (Gabriel). However, for one participant who did not need a voice reminder, this was experienced as stigmatising. | Creativity and customisation - allowing for tech to fit into everyday life - gives control. |
| CMO11: Familiar design | Peng et al., 2023, page 1067. | older people seem more likely to accept the relatively traditional healthcare product (i.e., blood pressure meter) which they are more acquainted when compared to the new emerging ones (e.g., smartwatch, GPS tracker, smart elderly home). The older population are generally considered resistant to change. They may lack the basic knowledge on new technologies, and thus are unwilling to use such technologies. | If older adults feel apprehensive towards technology (context), or where they lack knowledge on how to use the technology, telecare with a familiar design may make it easier for people to use, and reduce technological anxiety. |
| CMO12: User expectations | Hamblin et al., 2016, page 6. | a few in the study made negative comments about response arrangements when alerts were triggered, as they felt the responders took too long to reach them when they had activated their devices in emergencies; as a result, in two cases participants chose alternative providers. Inaccurate measurement with some devices could be off-putting (with bed sensors and, in particular, fall detectors cited as either too sensitive or not sensitive enough to record a ‘soft fall’) and led to their removal. | When technology fails to meet goals and expectations, it will lead to a person stopping use. |
